# A Sri Lankan infant with immunoglobulin resistant incomplete Kawasaki disease with a vesicular psoriasiform rash, hypertension and late onset small joint arthritis: a case report

**DOI:** 10.1186/s12887-022-03510-z

**Published:** 2022-07-23

**Authors:** Thabitha Jebaseeli Hoole, Arjuna Salinda Athapathu, Anoma Damayanthi Abeygunawardene

**Affiliations:** 1 Post Graduate Institute of Medicine, Colombo, Sri Lanka; 2 Department of Paediatrics, Faculty of Medicine, University of Kelaniya, Ragama, Sri Lanka; 3 District General Hospital, Gampaha, Sri Lanka

**Keywords:** Incomplete Kawasaki disease, Vesicular guttate psoriasiform rash, Late onset arthritis, Hypertension, Infliximab

## Abstract

**Background:**

Kawasaki disease (KD) is a medium and small vessel vasculitis which usually has a good response to immunoglobulin therapy (IVIG). We present a case of incomplete KD with IVIG resistance associated with an unusual combination of vesicular guttate-psoriasiform rash, hypertension and late onset small joint arthritis.

**Case presentation:**

A four-month-old male infant from Sri Lanka presented with high fever, conjunctival redness, pedal oedema and skin rash. He was found to have hypertension since admission with a high white cell count and high inflammatory markers. There was poor response to intravenous antibiotics and subsequent 2D echocardiogram revealed coronary artery aneurysms suggestive of KD. In the third week of illness he developed a vesiculo-papular rash involving face, trunk and limbs – which on biopsy revealed features of guttate psoriasis.

Fever spikes continued and the coronary arteries showed progressive dilatation despite timely intravenous immunoglobulin administered on day 6 and methylprednisolone administered on day 10-13. Therapeutic response by means of reduction of fever was seen only after initiation of intravenous infliximab on day 28 of illness for which the fever responded within 24 hours. He developed a small joint arthritis of hands and feet on day 40 of illness which responded only after initiating methotrexate therapy. The hypertension persisted for 4 months after the onset of the illness before complete resolution.

**Conclusion:**

This case report depicts an unusual presentation of KD with a vesicular guttate-psoriasiform eruption, hypertension and late onset small joint arthritis. It highlights that clinicians should be aware of the fact that KD could present with such atypical manifestations and could develop unusual complications.

## Background

Kawasaki Disease (KD) is a medium and small vessel vasculitis with a predilection for the coronary arteries. Around 20-25% of untreated children develop coronary artery aneurysms, while less than 5% children treated with immunoglobulin do so [1]. The characteristic rash seen in KD is a scarlatiniform or erythema multiforme-like morbilliform polymorphous exanthem [2]. We present an infant who had an unusual combination of guttate-psoriasiform vesicular eruption, hypertension and late onset small joint arthritis associated with incomplete KD, who went on to develop coronary artery aneurysms despite early initiation of immunoglobulin therapy, eventually needing methylprednisolone, methotrexate and infliximab.

## Case presentation

A four-month-old previously well Sri Lankan male infant from an urban area presented with high grade fever of 102-103 °F of 2 days duration. He had conjunctival redness, bilateral pedal oedema, a scaly rash in the cheeks, and a sand-paper like papular erythematous rash on the trunk and bilateral upper and lower limbs. Both liver and spleen were palpable 2 cm below the costal margin. There was no redness in the tongue and no cervical lymphadenopathy. Other system examinations were normal, except for high blood pressure measured by both manual and electronic methods, which was above the 99th percentile.

The initial full blood count showed a total white cell count (WBC) of 18,000/uL with a neutrophil leukocytosis of 53%, a platelet count of 552,000/uL and a haemoglobin of 12 g/dL. C-reactive protein (CRP) was 130 mg/dL and the erythrocyte sedimentation rate (ESR) was 10 mm in the 1st hour. Serum sodium was 134 mmol/L and potassium 5.4 mmol/L. Renal and liver function tests were normal. Covid-19 rapid antigen and PCR tests were negative.

He was empirically initiated on intravenous cefotaxime and flucloxacillin based on the local sensitivity patterns, suspecting either a streptococcal or staphylococcal septicaemia. However, blood culture was sterile and high fever spikes continued despite continuous antibiotics. Ultrasound scan abdomen on day four was normal and 2D echocardiogram did not show any coronary artery dilatations. Atypical Kawasaki disease was suspected and intravenous immunoglobulin (IVIG) 2 g/kg was administered on day six, followed by aspirin 80 mg/kg/day in four divided doses. As the response was poor, investigations were repeated. CRP and ESR had risen to 143 mg/dL and 130 mm/1st hour respectively. Cerebrospinal fluid (CSF) showed polymorphs 10/mm^3^, lymphocytes 43/mm^3^, red cells 23/mm^3^ and protein 136 mg/dL. Antibiotics were escalated to intravenous meropenam and vancomycin. CSF culture showed no growth.

Fever spikes continued on day ten and the child developed periungual desquamation in fingers and toes. 2D echocardiogram was repeated which showed dilated coronary arteries (left main coronary artery 5.3 mm, left anterior descending artery 6.5 mm, right coronary artery 5 mm) and a thin pericardial effusion suggestive of KD. A second dose of IVIG 2 g/kg was given and Aspirin dose increased to 100 mg/kg/day. Intravenous Methylprednisolone 30 mg/kg pulse therapy was given for 3 days followed by oral prednisolone. Fever settled for 72 hours, only to recur again. Repeat 2D echocardiogram on day fifteen showed progressive worsening of the coronary dilatation (LMCA 5.7 mm, LAD 9 mm, RCA 6 mm). Electrocardiogram (ECG) showed ST elevations in inferior leads which were persistent on serial ECGs. Cardiac Troponin I levels and Creatine-kinase levels were normal. Clopidogrel and warfarin were added while Aspirin was continued. Oral prazosin and furosemide were added to control the hypertension. His serum electrolytes, renal functions and ultrasound abdomen and kidney-ureter-bladder and renal artery doppler were normal. Extensive investigations in view of aetiology for hypertension such as renal angiogram, urinary and plasma metanephrines, renin-aldosterone levels were not performed as the preliminary investigations were normal and due to limitation of resources in the local setting. His 2D-Echocardiogram did not reveal left ventricular hypertrophy and ophthalmic assessment did not reveal hypertensive retinopathy which confirmed the acute onset of hypertension with the current illness.

**Fig. 1:**
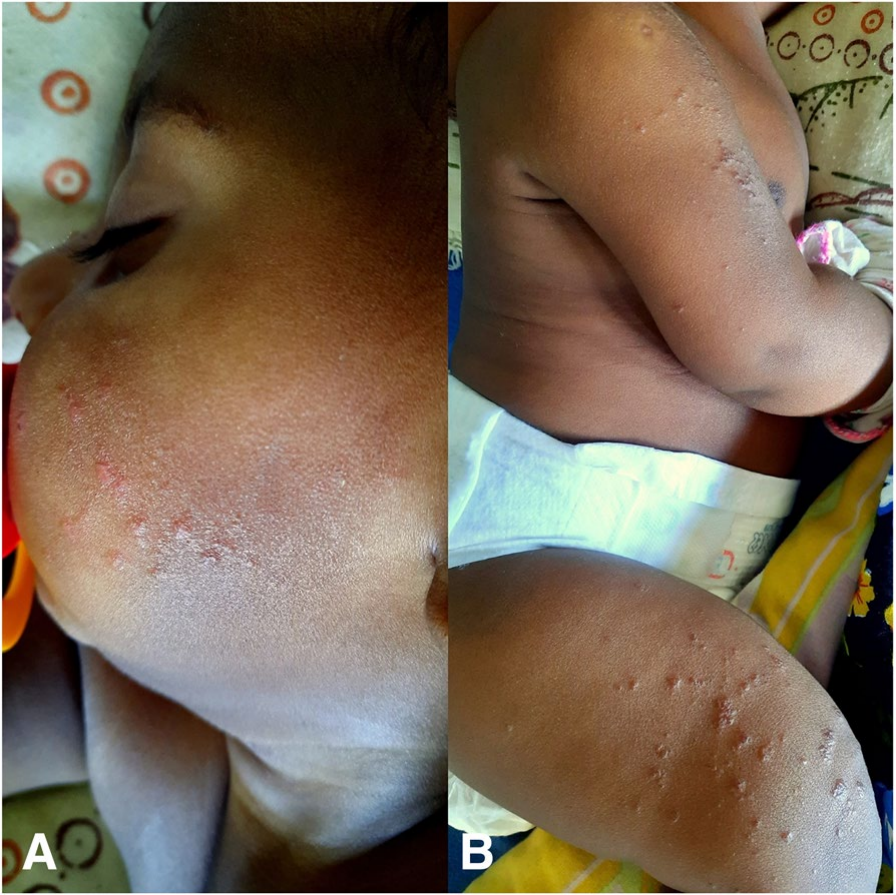
Photograph of the child taken during the second week of illness showing vesiculo-papular rash involving **A** Face and **B** Upper and lower limbs

**Fig. 2:**
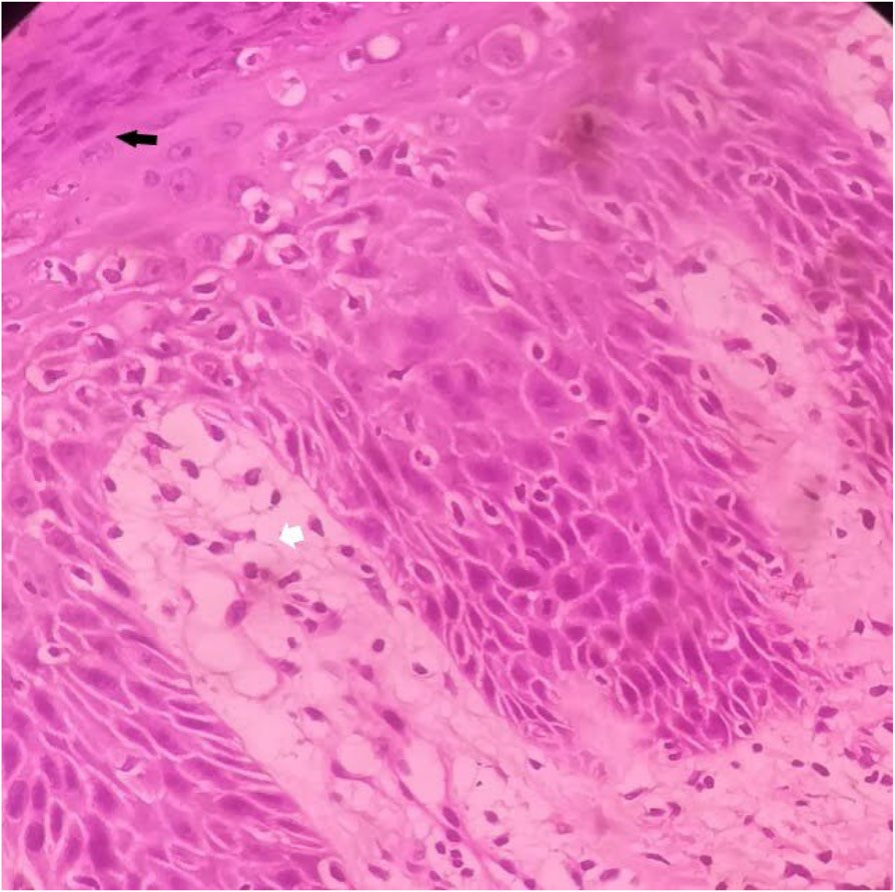
Skin biopsy showing parakeratosis (black arrow) and neutrophils in the epidermis (white arrow) with broad papillae suggestive of guttate psoriasis

On day sixteen he developed a vesiculo-papular rash involving face, trunk and distal upper and lower limbs including the periungual regions and the perineum (Fig. 1). It progressively evolved into bullous lesions. Biopsy of the rash revealed parakeratosis and neutrophils in the epidermis with broad papillae suggestive of guttate psoriasis (Fig. 2). It did not show small or medium vessel vasculitis.

**Fig. 3:**
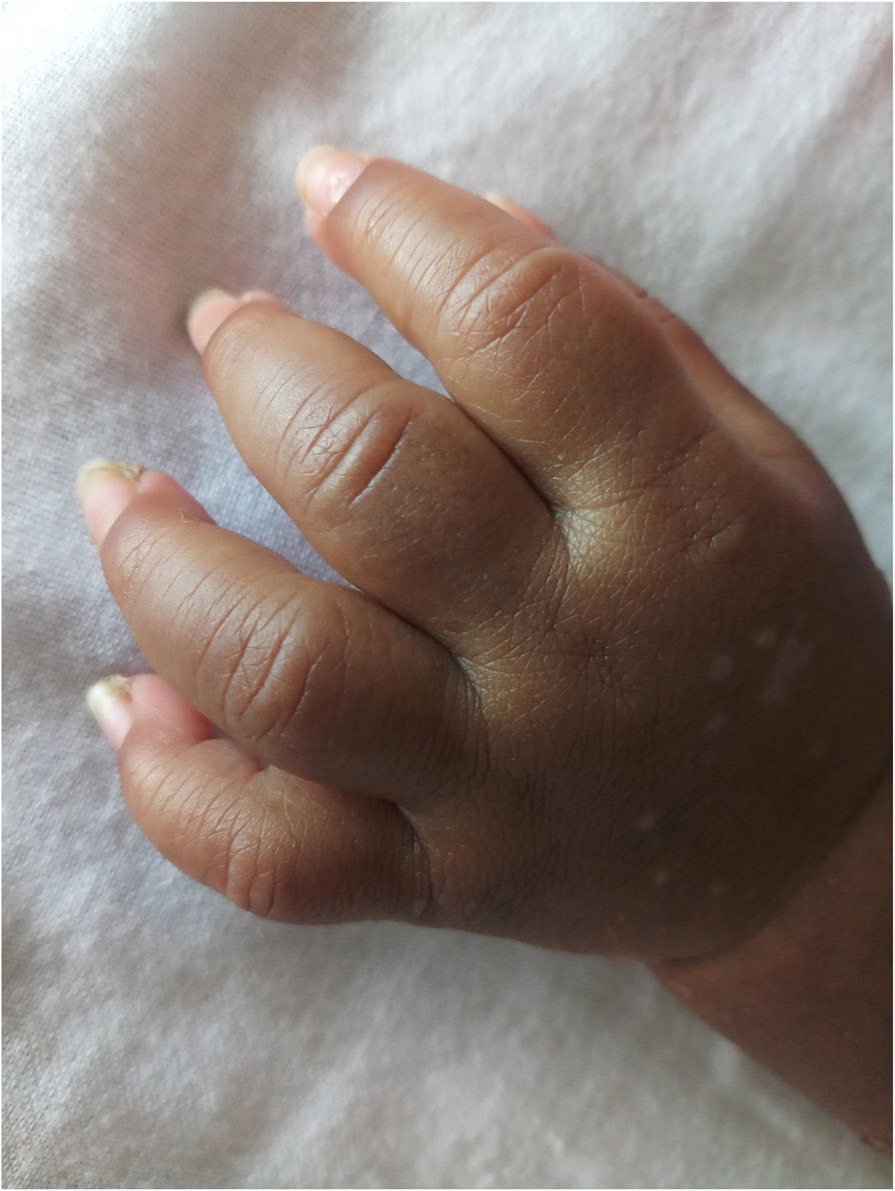
Swelling of middle interphalangeal joints of the left upper limb

Intravenous infliximab 5 mg/kg was administered on the 28th day of illness after screening for and excluding tuberculosis, followed by two more doses on the 2nd and 6th week after the initial dose. Fever responded to infliximab within 24 hours and the skin lesions showed gradual improvement. He developed a small joint arthritis involving proximal and middle inter-phalangeal joints of hands and feet on day 40 which showed a diurnal worsening (Fig. 3). Oral Methotrexate was added. Repeat 2D echocardiogram on day 60 showed a reduction in the coronary artery diameters, finally indicating a therapeutic response (LMCA 5.3 mm, LAD 6.9 mm, RCA 5 mm). The infant was discharged on day 61 of illness on aspirin, warfarin and prazosin. Oral prednisolone, clopidogrel and methotrexate were gradually tailed off after discharge. Oral prednisolone 2 mg/kg dose was given for 6 weeks followed by gradual taper over a period of 3 months.

On follow up, his arthritis and skin rash resolved completely. The hypertension which was present from the beginning of illness settled completely four and a half months from disease onset, enabling discontinuation of anti-hypertensive medications.

## Discussion and conclusion

Kawasaki Disease (KD) is a self-limiting medium and small vessel vasculitis lasting for an average of twelve days without treatment [3]. Coronary artery involvement can lead to significant morbidity and mortality.

The diagnosis of KD is based on a set of clinical criteria including fever lasting for 5 or more days along with at least four of the five physical findings [3]. Patients who fail to fulfil the full diagnostic criteria, but suspected to have the disease are said to have incomplete Kawasaki disease. These patients have similar susceptibility to coronary sequelae [4]. Incomplete KD should also be suspected in infants less than 6 months of age with unexplained fever lasting 7 days or more, even if they have no clinical findings of KD [3]. Our infant had persistent fever, skin rash and peripheral edema which were supportive of incomplete KD.

The well-described skin rash seen in KD is a generalized erythematous polymorphous exanthem. Other kinds such as scarlatiniform, urticarial, erythrodermatous, or micro-pustules have been described [2]. However bullous or vesicular rashes have not been reported prior. Our infant had an erythematous rash at the onset which evolved into vesiculo-bullous lesions. The skin biopsy revealed changes suggestive of guttate psoriasis. Psoriasiform rashes have been described in association with KD [5–8]. It has been postulated that a common pathogenic mechanism possibly induced by superantigens may have a role in the pathophysiology [7].

Another unusual finding in our infant was persistent hypertension that required treatment. Hypertension in KD can be due to the vasculitis itself, due to renal artery involvement causing stenosis or due to drug therapy with steroids [9, 10]. Our infant had hypertension from the onset of the disease, making steroids an unlikely culprit, but steroids could have contributed to the persistence of the hypertension later on. There has been a similar case in an infant with KD with idiopathic hypertension [11]. Hypertension in KD may in fact be much more common than reported in literature due to the fact that it is commonly overlooked and considered to be due to irritability and restlessness associated with the disease [11]. The exact pathophysiology of hypertension in KD remains to be elucidated.

The prevalence of arthritis in KD has reduced significantly during the immunoglobulin era [12, 13]. Arthritis can be early or late onset. Early onset type occurs during the first 7-10 days of illness and tends to involve multiple joints such as small interphalangeal joints as well as large weight-bearing joints. Late-onset type usually develops on the 10th to 14th day of illness or later, and affects the knees and ankles, and lasts up to 6 - 8 weeks [12]. Our patient had a late onset arthritis, but it mainly involved interphalangeal joints. Some studies suggest that joint involvement is associated with increased IVIG resistance [12].

The mainstay of treatment for KD are immunoglobulin and aspirin. IVIG resistance is known to be associated with an increased risk of coronary anomalies [14]. It is defined as persistent fever of any magnitude 24-36 hours after completion of IVIG therapy or return of fever of any magnitude after an afebrile period not explained by any cause other than KD up to 2 weeks after starting treatment [14]. Several other options such as adjunctive steroids and biological disease modifying agents such as infliximab are used as second line agents [15, 16]. Studies have shown that TNF-α receptor antagonists such as infliximab might reduce treatment resistance but their effect in reducing coronary artery abnormalities was not clear [17, 18]. Treatment with cyclosporine or methotrexate may be considered in those patients who fail multiple doses of IVIG and steroids, but larger randomized trials are still not available [19].

Our infant developed coronary artery aneurysms despite early initiation of treatment. The clinical features including fever, psoriasiform rash and the coronary artery dilatations responded only after initiating infliximab therapy. Arthritis responded to methotrexate .

This case report describes an unusual combination of vesicular guttate-psoriasiform rash, hypertension and late-onset small joint arthritis in association with KD which ultimately needed intravenous infliximab. It highlights that clinicians should be aware of the fact that KD could present with such atypical manifestations and could develop unusual complications.

## Data Availability

Not applicable.

## Abbreviations

KD: Kawasaki Disease
IVIG: Intravenous Immunoglobulin
LMCA: Left Main Coronary artery
LAD: Left Anterior Descending Artery
RCA: Right Coronary Artery
